# Construction and Evaluation of a Subcutaneous Splenic Injection Port for Serial Intraportal Vein Cell Delivery in Murine Disease Models

**DOI:** 10.1155/2019/5419501

**Published:** 2019-05-02

**Authors:** Toshio Miki, Chika Takano, Irving M. Garcia, Brendan H. Grubbs

**Affiliations:** ^1^Department of Surgery, Keck School of Medicine, University of Southern California, 2011 Zonal Avenue, HMR 509A, Los Angeles, CA 90033-9141, USA; ^2^Department of Obstetrics and Gynecology, Keck School of Medicine, University of Southern California, 1200 N. State Street, IRD 220, Los Angeles, CA 90033, USA

## Abstract

The liver is the largest internal organ and the center of homeostatic metabolism. Liver-directed cell transplantation is, therefore, an attractive therapeutic option to treat various metabolic disorders as well as liver diseases. Although clinical liver-directed cell transplantation requires multiple cell injections into the portal venous system, a mouse model is lacking which allows us to perform repetitive cell injections into the portal venous system. Here, we propose a surgical model that utilizes the spleen as a subcutaneous injection port. Mouse spleens were translocated under the skin with intact vascular pedicles. Human placental stem cell transplantations were performed one week following this port construction and repeated three times. Cell distribution was analyzed by quantifying human DNA using human Alu-specific primers. About 50% of the transplanted cells were located homogeneously in the liver one hour after the splenic port injection. Fluorescent-labeled cell tracking and antihuman mitochondrion immunohistochemistry studies demonstrated that the cells localized predominantly in small distal portal branches. A similar cell distribution was observed after multiple cell injections. These data confirm that the subcutaneous splenic injection port is suitable for performing repetitive cell transplantation into the portal venous system of mouse models.

## 1. Introduction

Hepatocyte transplantation is one of the promising regenerative approaches for the restoration of or compensation for impaired liver functions [1–3]. Although the therapeutic efficacy of this approach has been demonstrated in over 100 clinical trials, limited availability of the human hepatocyte prohibits the use of this therapeutic option for patients with liver diseases ranging from congenital metabolic disorders to liver cirrhosis [4, 5]. Recent advancements in stem cell biology have suggested that stem cell-derived hepatocyte-like cells can be an attractive alternative to the use of scarce human hepatocytes [6, 7].

As stem cell technology has advanced, there has been an increased demand for a suitable rodent model to perform preclinical liver-directed cell transplantation studies. When used clinically for the treatment of human disease, hepatocytes are transplanted multiple times into the portal circulation in order to obtain a therapeutic dose. This is accomplished via a catheter placed in either the intrahepatic portal vein, the middle colic vein, the inferior mesenteric vein, or the patent umbilical vein in the case of neonatal recipients. However, due to size limitations, cell injection routes are limited in rodent models. The size of the mouse mesenteric vein and the difficulty of obtaining hemostasis at this site prohibit its use as an injection site. Although direct liver injection can be used in hairless neonatal mouse models, the direct liver injection approach cannot avoid the possibility of injecting cells into the hepatic venous system, and obtaining hemostasis can be difficult. This makes intrasplenic cell injection the current “gold standard” procedure for cell transplantation in rodent models. Intrasplenic cell injection also minimizes the risk of causing an increase in portal pressure as the spleen can serve as a pressure buffer zone.

Here, we report a surgical procedure to mobilize the mouse spleen into a subcutaneous pocket that was utilized as a subcutaneous injection port for the performance of repeated intraportal venous cell injections. The in vivo distribution of these transplanted cells was subsequently evaluated in this mouse model.

## 2. Materials and Methods

### 2.1. Mice

All mice used in this study were bred and euthanized appropriately following the protocols that were approved by the University of Southern California Institutional Animal Care and Use Committee and conducted following the NIH Guide for the Care and Use of Laboratory Animals. Breeder heterozygous pairs of NOD.129(B6)-Prkdc^scid^ Idua^tm1Clk^ mice were obtained from The Jackson Laboratory (#004083), housed under specific-pathogen-free conditions and provided with regular chow (TEKLAD #2018) and sterile/acidified water. PCR-based genotyping was performed with specific primers according to The Jackson Laboratory's instructions.

### 2.2. Subcutaneous Splenic Injection Port Construction

**Figure 1:**
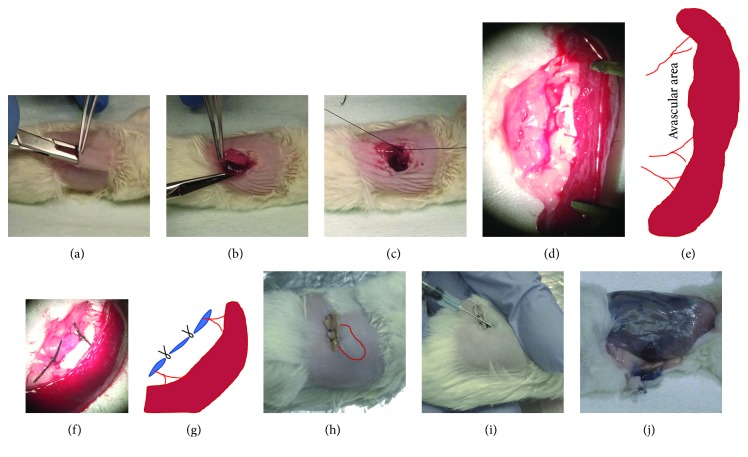
Construction of the subcutaneous splenic injection port. (a) After hair removal from the surgical area, a subcutaneous pocket was created with a straight hemostat. (b) The spleen was translocated into the subcutaneous pocket. (c) The muscle layer was closed deep to the spleen. (d) Macro view of the translocated spleen. (e) The traced illustration indicates the avascular area of the splenic vascular pedicle. (f) Macro view of the closed muscle layer. (g) The traced illustration depicts the closure of the muscle layer supporting the spleen in the developed subcutaneous pocket. (h) One week following surgery. (i) hAECs were resuspended in 50% trypan blue/PBS solution and transdermally injected into the spleen. (j) One hour after the cell injection, no leakage was observed in the pocket.

One week prior to planned cell injections, the mouse splenic port was prepared by the following technique. Briefly, the hair of the left upper back and trunk area was shaved and chemically removed using depilatory cream. A skin incision was then made over the spleen, and a subcutaneous pocket was then prepared with a straight hemostat (Figure 1(a)). The muscle layer was opened over the spleen to safely access to the gastrosplenic ligament, connecting the upper pole of the spleen and the stomach. The spleen was mobilized and translocated to the subcutaneous pocket (Figure 1(b)), and the muscle layer was subsequently closed over the avascular area between splenic vessels to keep the spleen in the subcutaneous pocket (Figures 1(c)–1(g)). Finally, the skin was closed with 6-0 sutures. Of note, the initial skin incision was relatively larger than what is normally required for a simple splenic cell injection, as it allowed better exposure for identification of the intraperitoneal anatomy.

### 2.3. Preparation of Fluorescent-Labeled Immortalized Human Amniotic Epithelial Cell Lines

Primary human amniotic epithelial cells (hAECs) from five different donors were immortalized using a SV40 Lentiviral vector (pLenti-SV40-T+t, Applied Biological Materials Inc., Richmond, BC, Canada). One line (iAE124) was selected and used for further lentiviral GFP labeling (PL-SIN-EF1a-EGFP).

### 2.4. Cell Transplantation

One week following construction of the subcutaneous splenic injection port, cell injection was performed using the GFP-positive immortalized hAECs (iAE124-GFP) and primary hAECs. A total of 1.5 million cells were suspended in 200 *μ*l of 50% trypan blue/PBS solution. The recipient mice were anesthetized with isoflurane inhalation. The subcutaneous spleen was visible and palpable underneath the skin (Figure 1(h)). Intrasplenic cell injection was performed through the skin at a 400 *μ*l/min injection speed. The needle was aimed at the lower pole of the spleen, and the needle tip was inserted into the middle of the spleen (Figure 1(i)). After the cell injection, the needle was removed without additional hemostatic measures. Multiple cell injections were tested with three mice. Each mouse received primary hAECs of 1.5 million cells per injection for 3 times one week apart. One week after the last primary hAEC injection, iAE124-GFP cells were injected to confirm the function of the subcutaneous splenic injection port.

### 2.5. Human DNA Quantification

**Figure 2:**
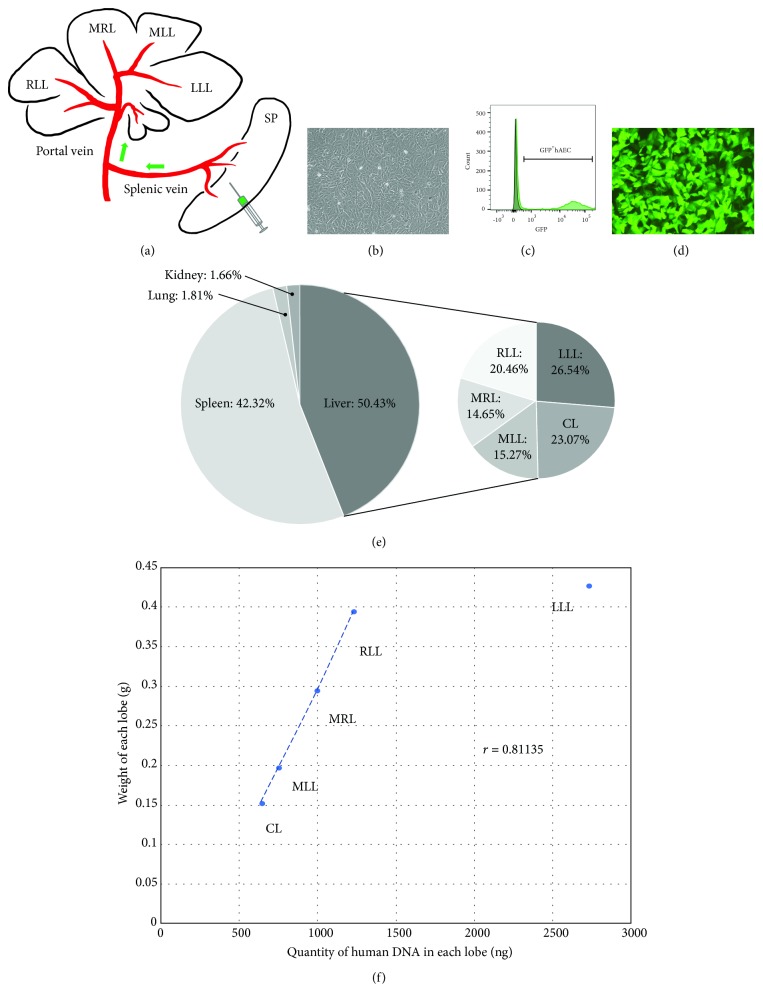
Cell distribution following intrasplenic cell injection. (a) Illustration of mouse liver lobes and flow of the injected cells via the splenic vein to branches of the portal vein: left lateral lobe (LLL), middle left lobe (MLL), middle right lobe (MRL), right lateral lobe (RLL), and caudate lobe (CL). (b) Phase-contrast image shows the morphology of the immortalized human amniotic epithelial cells (hAECs). (c) The histogram of FACS prior to sorting. (d) Fluorescent image demonstrating the green fluorescent protein- (GFP-) labeled immortalized hAECs after sorting. (e) Cell distribution pie chart. The left chart demonstrates the ratio of human DNA to total DNA from each tissue. The right chart demonstrates the ratio of adjusted human DNA quantity in each liver lobe. (f) A dot plot demonstrating the correlation of the size of each lobe and adjusted quantity of human DNA in each lobe.

One hour after cell injections were performed, the animals were euthanized and the cell injection site and surrounding tissue were examined for the trypan blue staining (Figure 1(j)). The liver, lung, kidneys, and spleen were harvested for histological analyses and human DNA quantification. The liver was dissected, and each lobe was identified and separated: the left lateral lobe (LLL), middle left lobe (MLL), middle right lobe (MRL), right lateral lobe (RLL), and caudate lobe (CL) (Figure 2(a)). DNA was isolated with a genomic DNA isolation kit (ZR-96 Quick-gDNA, Zymo Research, Irvine, CA, USA) from each entire lobe and whole lung, kidney, and spleen tissues. To quantify human DNA, Alu elements, an A-rich region formed after the evolutionary divergence of rodents and humans, were detected by using real-time quantitative PCR (qPCR). The intra-Alu Yb8 primers (forward: 5′-CGA GGC GGG TGG ATC ATG AGG T-3′ and reverse: 5′-TCT GTC GCC CAG GCC GGA CT-3′) were used with the SYBR green system [8]. The sensitivity of this primer set is 0.01%. A total of four animals were examined (*n* = 4).

### 2.6. Human Amniotic Epithelial Cell (hAEC) Detection in the Mouse Liver

In order to detect hAECs in the recipient mouse liver, two cell identification methods, fluorescent-labeled cell tracing (GFP-positive hAEC injection) and antihuman mitochondrion immunohistochemical staining, were used.

Recipient livers were sliced at 5 mm thickness and immersed in 4% paraformaldehyde/PBS fixation buffer overnight at 4°C. The samples were divided in two groups for cryosection and for paraffin embedding/section. The cryosection samples were embedded in an OCT compound and sectioned at 8 *μ*m thickness. The sections were briefly washed with PBS and mounted with antifade mounting medium with DAPI (Invitrogen). Liver structures were visualized using Alexa Fluor 594 Phalloidin (Invitrogen) as a counterstain. The paraffin sections were stained with an anti-human mitochondrion antibody (MAB1273, clone 113-1, MilliporeSigma) at 1 : 300 dilution overnight at 4°C. The detection was performed with a peroxidase detection kit (ImmPRESS HRP reagent kit/goat anti-mouse IgG, Vector Laboratories) and peroxidase substrate (ImmPACT NovaRED, Vector Laboratories) by following the manufacturer's instruction. Histological analyses were performed using a fluorescent inverted microscope (Nikon TE-2000), and images were captured with NIS-Elements microscope imaging software.

### 2.7. Statistical Analysis

Results are expressed as mean ± standard error of the mean (SEM). Statistical analysis was performed with Prism 5.0a (GraphPad Software, San Diego, CA, USA). Experimental and control groups were compared with paired or unpaired one-way ANOVA (with Bonferroni post hoc analysis and Dunnett's multiple comparison). A value of *p* < 0.05 was considered statistically significant.

## 3. Results

### 3.1. Establish a Surgical Procedure to Utilize the Spleen as a Subcutaneous Injection Port

First, we have established a surgical procedure which involves detachment of the spleen from the stomach and its relocation to the subcutaneous pocket with an intact vascular pedicle. Unlike conventional splenic injections, a relatively large skin incision was required over the upper portion of the spleen. To access the gastrosplenic ligament, the greater curvature of the stomach was grasped and used for manipulation. To test the feasibility of this surgical procedure for future usage with disease mouse models, we used semi-immunodeficient Idua knockout mice as the recipients. Despite using this relatively fragile disease model mouse, there was no mortality associated with the procedure. One complication included a failure of skin closure due to the insufficient size of the subcutaneous pocket, suggesting that a generous subcutaneous pocket should be developed in order to have a sufficient space to accommodate the spleen (Figure 1(a)). To prevent the mobilized spleen from falling back to its physiologic position in the abdominal cavity, the muscle layer was sutured at the middle avascular area of the splenic hilum (Figures 1(e)–1(g)). The total surgery time was about 10 minutes. After one week, the incision remained closed and was almost completely healed (Figure 1(h)). The subcutaneous spleen was visible and palpable. We have tested different gauge size needles from 30G to 25G. The larger needle would be preferred to decrease the impact of shear stress on the cells. The 25G needle did not cause any hemostatic or leakage problems (Figure 1(i)). The trypan blue dye injection confirmed there was no leakage one hour following injection of the 200 *μ*l cell suspension injection (Figure 1(j)). Despite the size of the relatively large subcutaneous pocket, the spleen was tightly encapsulated without excessive subcutaneous dead space. As expected, the skin layer functioned as a sealer as is seen in other common intravenous injections to prevent bleeding without the need for a hemostatic procedure.

### 3.2. Fluorescent-Labeled Immortal Human Amniotic Epithelial Cells

Primary human amniotic epithelial cells (hAECs) from five different donors were immortalized using a SV40 Lentiviral vector (pLenti-SV40-T+t, Applied Biological Materials Inc., Richmond, BC, Canada). The established immortalized hAEC lines were morphologically evaluated (Figure 2(b)). One line (iAE124) was selected and used for further lentiviral GFP labeling (PL-SIN-EF1a-EGFP). GFP-positive cells were subsequently isolated by fluorescence-activated cell sorting (FACS) and used to establish a GFP-positive immortalized hAEC line (iAE124-GFP) (Figures 2(c) and 2(d)).

### 3.3. In Vivo and Intrahepatic Cell Distribution

To evaluate the in vivo cell distribution following splenic reservoir cell injections, the presence of human DNA was quantified by qPCR using human Alu sequence-specific primers [8]. The spleen, the liver, the lung, and the kidneys were harvested, and DNA was isolated from each of these organs. In consideration of blood flow patterns, we considered the lung as a near sentinel point of cell leakage from the liver, and the kidneys were used to detect any systemically circulating injected human cells. One hour following cell injections, 42.32 ± 14.64% of detected human DNA was found in the spleen and 50.43% was in the liver (Figure 2(e)). In most of the cases, human DNA levels in lung samples were at or under the borderline detection level (<0.01%). However, in one case, we detected 0.51% human DNA from the lung sample. This finding indicates that the injected cells can leak from the intrahepatic portal system into the hepatic venous system.

We further investigated the detailed intrahepatic cell distribution by dissecting the liver lobes. The quantity of human DNA in each liver lobe was estimated, and the intrahepatic cell distribution ratio was calculated (Figure 2(e)). The left lateral lobe (LLL) and caudate lobe (CL) showed the presence of a relatively higher number of the transplanted cells; however, there were no statistically significant differences. Although individual differences were observed, the overall quantity of human DNA in each lobe correlated with the wet weight of each lobe (*r* = 0.81135) (Figure 2(f)). This data indicates that the cell distribution follows the liver hemodynamics and is equally distributed throughout the liver.

**Figure 3:**
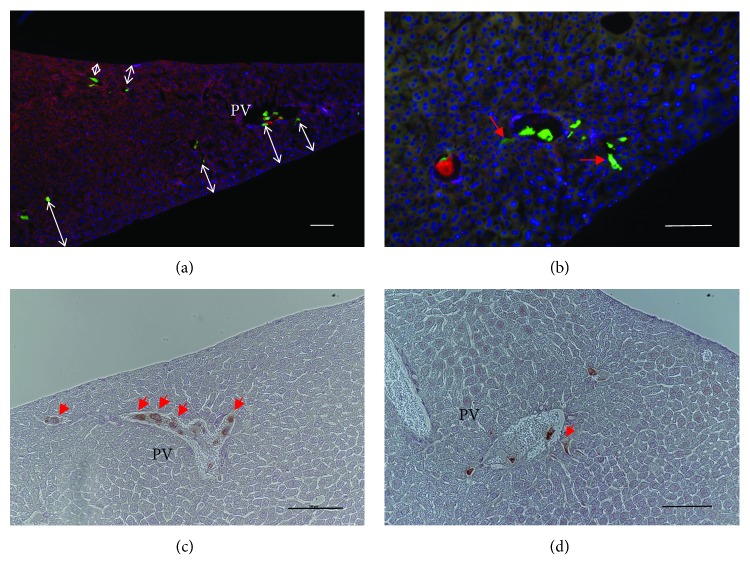
GFP-labeled immortalized hAEC injection. (a) The low magnification fluorescent image (×10) of a recipient liver one hour after cell injection. (b) The high magnification fluorescent image revealed GFP-positive cells in the liver parenchyma (red arrow). DAPI (blue) and Alexa Fluor 594 Phalloidin (red) were used as counterstains to visualize the nuclear and liver structures, respectively. (c) Antihuman mitochondrion immunohistochemistry demonstrates cell distribution and microemboli in the mouse portal vein. (d) Antihuman mitochondrion immunohistochemistry demonstrates human cells in the mouse liver parenchyma.

GFP-positive cell tracing and antihuman mitochondrion immunohistochemistry demonstrated that the cells were heterogeneously located within each of the lobes. The GFP-positive cells were found predominantly in small distal portal branches at an average distance from the liver surface of 111.04 ± 59.14 *μ*m (Figure 3(a)). Some cells were observed in the periportal region of the relatively large portal veins, while some cells formed cell aggregates (microemboli) in the intrahepatic portal capillaries. After one hour following cell injection, some cells were already located in the liver parenchyma (Figure 3(b)). A similar cell distribution pattern was observed with antihuman mitochondrion immunohistochemical analyses. Human mitochondrion-containing cells were observed in the intrahepatic portal capillaries (Figure 3(c)) as well as the periportal region (Figure 3(d)).

### 3.4. Multiple Cell Injection

**Figure 4:**
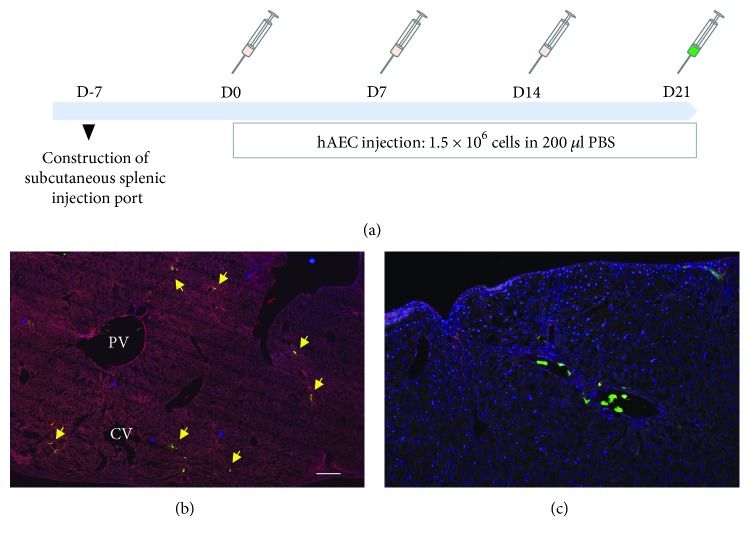
Multiple human amniotic epithelial cell injections. (a) The schematic shows an overview of the multiple cell injection study design. One week before the first cell transplantation, the subcutaneous cell injection port construction surgery was completed. A total of 1.5 million hAECs were transplanted every week for four times. The final cell injection was performed with GFP-labeled hAECs to confirm the function of the splenic injection port. (b) The low magnification fluorescent image (×10) of the recipient liver one hour after the final GFP-positive cell injection. The yellow arrows indicate the cells located in the area of small distal portal branches. (c) The cell distribution and migration are similar to those of the first cell transplantation after serial cell injections. DAPI (blue) and Alexa Fluor 594 Phalloidin (red) were used as counterstains to visualize nuclear and liver structures, respectively.

The function of the subcutaneous splenic cell injection port was confirmed after multiple hAEC transplantations by injecting GFP-positive hAECs. The subcutaneous spleen was clearly visible under the skin and palpable one month after the surgery. Following three injections of non-GFP-labeled hAECs at one week intervals, GFP-positive immortalized hAECs were injected (Figure 4(a)). The GFP-positive cells were detected in the recipient's liver (Figures 4(b) and 4(c)), and the cell number and distribution pattern were the same as those of single-injection cases. There was no evidence of leakage around the injection site, and no sign of inflammation.

## 4. Discussion

Repeated cell injections are often required to transplant a sufficient number of cells for disease phenotype improvement. Clinical hepatocyte transplantation studies indicate that about 100 million cells per kg body weight are required to replace the estimated 5-20% of the patient's missing enzyme function, necessary for phenotype stabilization/improvement [5]. For example, a 30 kg body weight infant requires three billion cells. To deliver such a large volume of cells in human patients, multiple cell transplantations are required. However, the current mouse intrasplenic cell injection methods are not suitable for the conduction of multiple cell transplantation procedures. Repetitive open surgery causes stress on the recipient mice and increases the risk of hemorrhage. In order to perform multiple cell injections in mouse models, we have established a minimally invasive surgical model that utilizes the spleen as a subcutaneous injection port. The results indicate that performance of multiple cell injections was feasible without the need for further surgeries.

In this study, we investigated the in vivo and intrahepatic cell distribution. Although one lung sample contained detectable human DNA, in most of the cases, the injected cells were retained in the spleen or the liver. The intrahepatic cell distribution was correlated with the size of each lobe. This data further supports the previous study using fluorescent beads which demonstrated that portal vein flow is evenly distributed to each lobe [9]. Using in vivo fluorescent microscopy, Timm et al. observed the formation of microemboli in small distal portal branches following injection of syngeneic hepatocytes. They also demonstrated heterogeneous portal venous perfusion which resulted in a highly heterogeneous cell distribution following intrasplenic injection [10]. While hepatic arterial flow homogeneously perfuses the liver acini, fluorescent dye injected into the portal venous system showed clear anatomical borders defining highly, semi-, and nonportal venous perfused liver acini. Due to the limited number of cells, we could not conclude whether the intralobular cell distribution is homogeneous or heterogeneous. However, we observed a tendency that the transplanted cells were identified predominantly in small distal portal branches at an average distance of about 100 *μ*m from the liver surface. Further investigation using this model will elucidate the cell distribution pattern and, if present, the mechanism resulting in heterogeneous cell distribution.

This model will be useful to optimize various preclinical therapeutic conditions using different disease model mice. We have reported the therapeutic efficacy of hAEC by percutaneous direct liver injection into a neonatal mouse model of mucopolysaccharidosis I [11]. Further optimization including cell dosing can be performed with this intrasplenic multiple cell transplantation model. Many disease model mice are sensitive and susceptible to physical agitation, making them unsuitable to undergo repetitive surgery or device implantation. Therefore, the subcutaneous splenic injection port could be useful to test both therapeutic biological and nonbiological products in these disease mouse models.

## 5. Conclusions

We have established a mouse model which allows for the minimally invasive performance of multiple cell injections. The cell distribution analyses indicated homogeneous cell distribution between the liver lobes, and heterogeneous distribution within the liver lobes. The subcutaneous splenic cell injection port was functional after multiple cell injections. This model will be useful to simulate clinical hepatocyte transplantation and facilitate preclinical studies using stem cell-derived hepatic cell transplantation studies.

## Data Availability

The data used to support the findings of this study are available from the corresponding author upon request.

## Abbreviations

GFP: Green fluorescent protein
FACS: Fluorescence-activated cell sorting
hAEC: Human amniotic epithelial cell
LLL: Left lateral lobe
MLL: Middle left lobe
MRL: Middle right lobe
RLL: Right lateral lobe
CL: Caudate lobe
OCT: Optimal cutting temperature
PBS: Phosphate-buffered saline
DAPI: 4′,6-diamidino-2-phenylindole
PCR: Polymerase chain reaction
TF: Tissue factors.
